# Association of “Ippatsu-Gei,” a Brief Psychosocial Interaction, with Spontaneous Activity and Adverse Events in Home-Visit Dysphagia Rehabilitation: A Retrospective Cohort Study

**DOI:** 10.3390/jcm15197745

**Published:** 2026-10-08

**Authors:** Chizuko Kameda, Kohei Yamaguchi, Miki Ishii, Ryosuke Yanagida, Kazuharu Nakagawa, Kanako Yoshimi, Haruka Tohara

**Affiliations:** Division of Gerontology and Gerodontology, Department of Dysphagia Rehabilitation, Institute of Science Tokyo, 1-5-45 Yushima, Bunkyo-ku, Tokyo 113-8519, Japan; chizuko59@gmail.com (C.K.); mickey.cookie05015@gmail.com (M.I.); ry.yanagida@gmail.com (R.Y.); nakagerd@tmd.ac.jp (K.N.); k.yoshimi.gerd@tmd.ac.jp (K.Y.); harukatohara@hotmail.com (H.T.)

**Keywords:** Ippatsu-gei, dysphagia, home-visit care, rehabilitation, spontaneous activity, psychosocial interaction

## Abstract

**Background/Objectives**: Direct and indirect training are well established in dysphagia rehabilitation, but the role of brief psychosocial interactions in home-based care remains unclear. This retrospective cohort study examined whether provider-based exposure to “Ippatsu-gei” was associated with spontaneous activity and adverse events during home-visit dysphagia rehabilitation. **Methods**: Clinical records were reviewed for patients who received at least one session between January and June 2019. Exposure was defined as care from dentists who routinely incorporated Ippatsu-gei into patient communication. Spontaneous-activity analysis required complete 180-day follow-up (*N* = 148); adverse-event analysis included patients with complete follow-up or a predefined event within 180 days (*N* = 150). **Results**: Spontaneous activity was documented in 18 of 148 patients (12.2%). Ippatsu-gei exposure was associated with higher odds of spontaneous activity (adjusted odds ratio 5.42; 95% confidence interval 1.62–18.18; *p* = 0.006), although the estimate remained imprecise. Adverse events occurred in 31 of 150 patients (20.7%) and were not significantly associated with Ippatsu-gei exposure (adjusted odds ratio 0.53; 95% confidence interval 0.20–1.39; *p* = 0.195). **Conclusions**: Provider-based Ippatsu-gei exposure was associated with retrospectively documented spontaneous activity, but causal interpretation is limited by temporal uncertainty, provider-related effects, and residual confounding. No statistically significant association with adverse events was observed.

## 1. Introduction

Dysphagia is a clinically significant condition that affects functional oral intake [1] and has been associated with reduced activity and impaired quality of life [2,3]. In community and home-based care settings, swallowing dysfunction has been associated with aspiration-pneumonia history [2,4] and can change over time in care-dependent patients [4]. Consequently, the goals of dysphagia rehabilitation in long-term care may extend beyond restoration of swallowing function to include maintenance of functional stability, support for daily activity and participation, and prevention of further decline.

Conventional dysphagia rehabilitation includes training, environmental adjustments, and dental interventions selected according to patient characteristics [5]. Psychosocial factors may also be relevant to activity, participation, and engagement in daily life. Previous studies have highlighted the importance of activity and participation in home rehabilitation among older adults [6], and behavioral activation has been investigated as an approach to promote engagement and well-being in older populations [7]. Spontaneity has also been examined in relation to psychological well-being [8]. In the present study, however, “spontaneous activity” was operationally defined as newly emerging, patient-initiated behaviors identified retrospectively from routine clinical records, such as going out, initiating exercise, eating out, traveling, or spending more time out of bed. It should therefore be regarded as a record-based outcome rather than a formally validated measure of motivation or spontaneity.

To our knowledge, no previous studies have specifically evaluated Ippatsu-gei in the context of dysphagia rehabilitation. Similar but not equivalent psychosocial approaches have been reported in rehabilitation and older-adult care across different cultural settings. Music- and singing-based interventions have been investigated in relation to swallowing difficulties [9], while humor, laughter, and culturally meaningful activities have been examined in relation to well-being and social functioning among older adults [10,11,12]. Laughter and social communication have also been examined in relation to oral frailty [13]. Interpersonal trust has been examined as an important component of patient–provider relationships [14]. Although these approaches differ substantially from Ippatsu-gei in their content, context, and purpose, they provide a broader psychosocial context in which culturally familiar forms of interpersonal engagement may be relevant to rehabilitation-related activity and participation.

“Ippatsu-gei” is a Japanese term referring to a brief, improvised performance intended to attract attention or create amusement within a social interaction. It is not an established or standardized medical or dysphagia rehabilitation technique. In the present clinical setting, Ippatsu-gei referred to brief, informal entertainment-based interactions incorporated pragmatically into routine patient communication by dentists who used such interactions in clinical practice. Examples included light humor, simple jokes, singing a familiar song, simple dance-like gestures, origami demonstrations, and basic magic tricks. The content, timing within the rehabilitation session, frequency, and duration of these interactions were not standardized and varied with the clinical context and the treating dentist’s judgment. The duration of individual interactions was not systematically documented. No age-based eligibility criterion was applied in the present study, and the analytic cohort ranged in age from 2 to 102 years.

In this retrospective cohort study, we evaluated whether provider-based exposure, defined as care from dentists who routinely incorporated Ippatsu-gei into patient communication, was associated with retrospectively documented spontaneous activity and the occurrence of adverse events during follow-up. Because exposure was provider-based and the timing and content of Ippatsu-gei were not standardized, the study was designed to evaluate associations observed in routine clinical practice rather than the causal effect of a standardized intervention.

## 2. Materials and Methods

### 2.1. Study Design and Setting

In this retrospective cohort study, we analyzed the clinical records of patients who received home-visit dysphagia rehabilitation provided by the Institute of Science Tokyo. Rehabilitation services were delivered by dentists and dental hygienists within a 16-km catchment area encompassing private residences and long-term care facilities, in accordance with Japanese medical regulations. In Japan, dental professionals play an important role in home-visit dysphagia rehabilitation, including oral management, swallowing assessment, and multidisciplinary rehabilitation [4]. Dentists may also perform videoendoscopic evaluation of swallowing (VE) as part of dysphagia management during home visits [4], although VE was not systematically performed in all participants in the present study.

During routine home visits, dentists conducted bedside swallowing assessments and provided individualized guidance based on each patient’s clinical condition. Dysphagia rehabilitation typically included posture adjustment, dietary modification, and basic swallowing exercises. In chronic home-based care, rehabilitation was not limited to physiological restoration of swallowing function but also included maintenance of functional stability and support for activity and participation. This approach is consistent with rehabilitation frameworks such as the International Classification of Functioning, Disability and Health (ICF), which recognize activity and participation as important components of rehabilitation [15].

All study procedures were conducted in accordance with the Declaration of Helsinki. The study is reported in accordance with the STROBE (Strengthening the Reporting of Observational Studies in Epidemiology) guidelines, and the completed checklist is provided as Checklist S1.

### 2.2. Participants

Clinical records from 1 January 2019 to 31 March 2020 were reviewed. Eligibility for the study cohort required receipt of at least one home-visit dysphagia rehabilitation session between 1 January and 30 June 2019. The index date was defined as the first home-visit dysphagia rehabilitation session in 2019.

Outcome-specific analytic cohorts were defined based on follow-up availability. For the spontaneous-activity analysis, complete follow-up for 180 days from the index date was required because incomplete follow-up could reduce the opportunity to ascertain newly emerging spontaneous activity. Patients without confirmed complete 180-day follow-up were therefore excluded from this analysis.

For the adverse-event analysis, patients were included if complete 180-day follow-up was available or if a predefined adverse event had been ascertained within the 180-day observation window before follow-up ended. Accordingly, patients with an adverse event confirmed within 180 days were retained even when complete 180-day follow-up was unavailable, because occurrence of the outcome within the prespecified period had already been established. Patients with incomplete follow-up and no ascertainable adverse event were excluded from the adverse-event analysis.

Missing data were not imputed.

### 2.3. Exposure: Ippatsu-Gei

The primary exposure was Ippatsu-gei, a brief, informal entertainment-based psychosocial interaction incorporated pragmatically into routine home-visit dysphagia rehabilitation. Ippatsu-gei was not an established or standardized medical or dysphagia rehabilitation technique. In routine clinical practice, it included culturally familiar, brief interactions such as light humor, simple jokes, singing a familiar song, simple dance-like gestures, origami demonstrations, and basic magic tricks.

The content, timing within the rehabilitation session, frequency, and duration of Ippatsu-gei were not standardized and depended on the clinical context and the treating dentist’s judgment. The duration of individual interactions was not systematically recorded in the clinical records.

Exposure classification was provider-based rather than based on session-level confirmation of individual Ippatsu-gei performances. Patients were classified as exposed if they received at least one home-visit dysphagia rehabilitation session during the observation period from dentists who routinely incorporated Ippatsu-gei into patient communication. Patients who did not receive care from such dentists during the observation period were classified as unexposed. Accordingly, the exposure variable reflected contact with providers who routinely used Ippatsu-gei and did not indicate that a standardized Ippatsu-gei interaction occurred during every rehabilitation session.

To account for heterogeneity in prior rehabilitation experience, prior rehabilitation exposure duration was calculated as the number of months between the first-ever home-visit dysphagia rehabilitation session and the index visit in 2019 (prior_exposure_months). This variable was modeled as a continuous covariate in the regression analyses.

### 2.4. Covariates

Baseline demographic and clinical characteristics were extracted from the earliest available record at or near the index visit. Variables included age, sex, residence (home or long-term care facility), Charlson Comorbidity Index (CCI) [16], Clinical Frailty Scale (CFS) [17], Functional Oral Intake Scale (FOIS) [1], and level of consciousness assessed using the Japan Coma Scale (JCS) [18]. Underlying clinical conditions, including nervous system disease, cerebrovascular disease, dementia, and physical injury, were also recorded.

Swallowing severity was characterized using the Penetration–Aspiration Scale (PAS) [19] among participants who underwent VE as part of routine clinical care. PAS is an 8-point scale describing the degree of airway invasion during swallowing, with higher scores indicating more severe airway compromise. Severe frailty was defined as CFS ≥6, and limited oral intake was defined as FOIS ≤3.

Covariates for the regression models were selected a priori on the basis of clinical relevance, previous literature, and model stability. Because the number of outcome events was limited, not all baseline characteristics showing between-group imbalance could be simultaneously included in the multivariable models.

### 2.5. Outcomes

The primary outcome was spontaneous activity, operationally defined as newly emerging, patient-initiated behaviors documented in routine clinical records during the 180-day observation period. Documented behaviors included going out, initiating exercise, eating out, traveling, and increased time spent out of bed. These behaviors were not prescribed as part of the rehabilitation program. Each patient was counted once, regardless of the number or type of spontaneous activities documented. Spontaneous activity was analyzed as a binary, record-based outcome and was not a formally validated measure of motivation or spontaneity.

Spontaneous-activity events were identified and classified retrospectively using this operational definition. The clinical-record review and classification were performed by a single investigator (C.K.), who was aware of each patient’s Ippatsu-gei exposure status at the time of outcome assessment. Therefore, outcome assessment was not blinded to exposure status.

Because exposure classification and spontaneous activity were both assessed within the same 180-day observation period and session-level timing of individual Ippatsu-gei interactions was not systematically recorded, the temporal sequence between Ippatsu-gei exposure and spontaneous activity could not always be established.

The secondary outcome was the occurrence of adverse events, defined as unplanned hospitalization, unplanned outpatient visits, or death within 180 days of the index date. Adverse events were analyzed as a binary outcome indicating the presence or absence of at least one predefined event during the observation window. Patients with an adverse event confirmed within 180 days were classified as having experienced the outcome even when complete 180-day follow-up was unavailable. Detailed outcome definitions, exposure classification, and analytical considerations are provided in Appendix A.

### 2.6. Statistical Analysis

Normality of continuous variables was assessed using the Kolmogorov–Smirnov test. Continuous variables are presented as mean ± standard deviation or median [interquartile range (IQR)], as appropriate. Categorical variables are presented as numbers (percentages). Between-group comparisons were performed using the Mann–Whitney U test for continuous variables and the chi-square test or Fisher’s exact test for categorical variables, as appropriate.

Absolute standardized mean differences (SMDs) were calculated to quantify the magnitude of baseline imbalance between the Ippatsu-gei and control groups. For prior rehabilitation exposure duration, the SMD was calculated using the underlying continuous values, although the variable is presented as median [interquartile range] in Table 1.

Associations between Ippatsu-gei exposure and each outcome were evaluated using univariate logistic regression followed by multivariable logistic regression. Covariates were selected a priori on the basis of clinical relevance, previous literature, and model stability.

For spontaneous activity, the multivariable model included Ippatsu-gei exposure, age, sex, CFS, and prior rehabilitation exposure duration. Because only 18 spontaneous-activity events were observed, model complexity was intentionally limited to minimize instability associated with the small number of outcome events.

For adverse events, the multivariable model included Ippatsu-gei exposure, age, sex, and prior rehabilitation exposure duration. CFS was not included in the final adverse-event model because its limited variability resulted in unstable and non-estimable coefficients in exploratory models.

Other baseline characteristics, including FOIS, dementia, and physical injury, showed between-group imbalance but were not simultaneously included in the multivariable models because of the limited number of outcome events and concerns regarding model stability. Consequently, residual confounding from measured and unmeasured baseline differences cannot be excluded.

Results are reported as odds ratios (ORs) or adjusted odds ratios (aORs) with 95% confidence intervals (CIs). A two-tailed *p*-value < 0.05 was considered statistically significant. Primary analyses were conducted using IBM SPSS Statistics for Windows, version 29.0 (IBM Corp., Armonk, NY, USA).

As a sensitivity analysis to reduce potential small-sample bias associated with the limited number of outcome events, Firth’s penalized logistic regression was performed using the same covariate specifications as the primary multivariable models. These analyses were conducted using R version 4.6.1 (R Foundation for Statistical Computing, Vienna, Austria) with the logistf package (version 1.26.1). Adjusted ORs with 95% profile penalized likelihood CIs and corresponding *p*-values are presented in Table S2.

### 2.7. Ethical Considerations

The Ethics Committee of the Institute of Science Tokyo approved this study (Approval No. D2023-027). The study was conducted in accordance with the Declaration of Helsinki. Given the retrospective design, the requirement for written informed consent was waived; however, study information was disclosed in accordance with institutional and national requirements. Clinical records were accessed for research purposes from 15 November 2023 onward. Data were anonymized before analysis, and the authors did not have access to directly identifiable personal information during or after data collection.

## 3. Results

### 3.1. Participant Flow and Baseline Characteristics

Clinical records from 1 January 2019 to 31 March 2020 were reviewed. Of these records, 309 patients were identified, and 37 patients with missing essential baseline data were excluded, leaving 272 patients for screening. Eligibility for the study cohort required at least one home-visit dysphagia rehabilitation session between 1 January and 30 June 2019.

**Figure 1:**
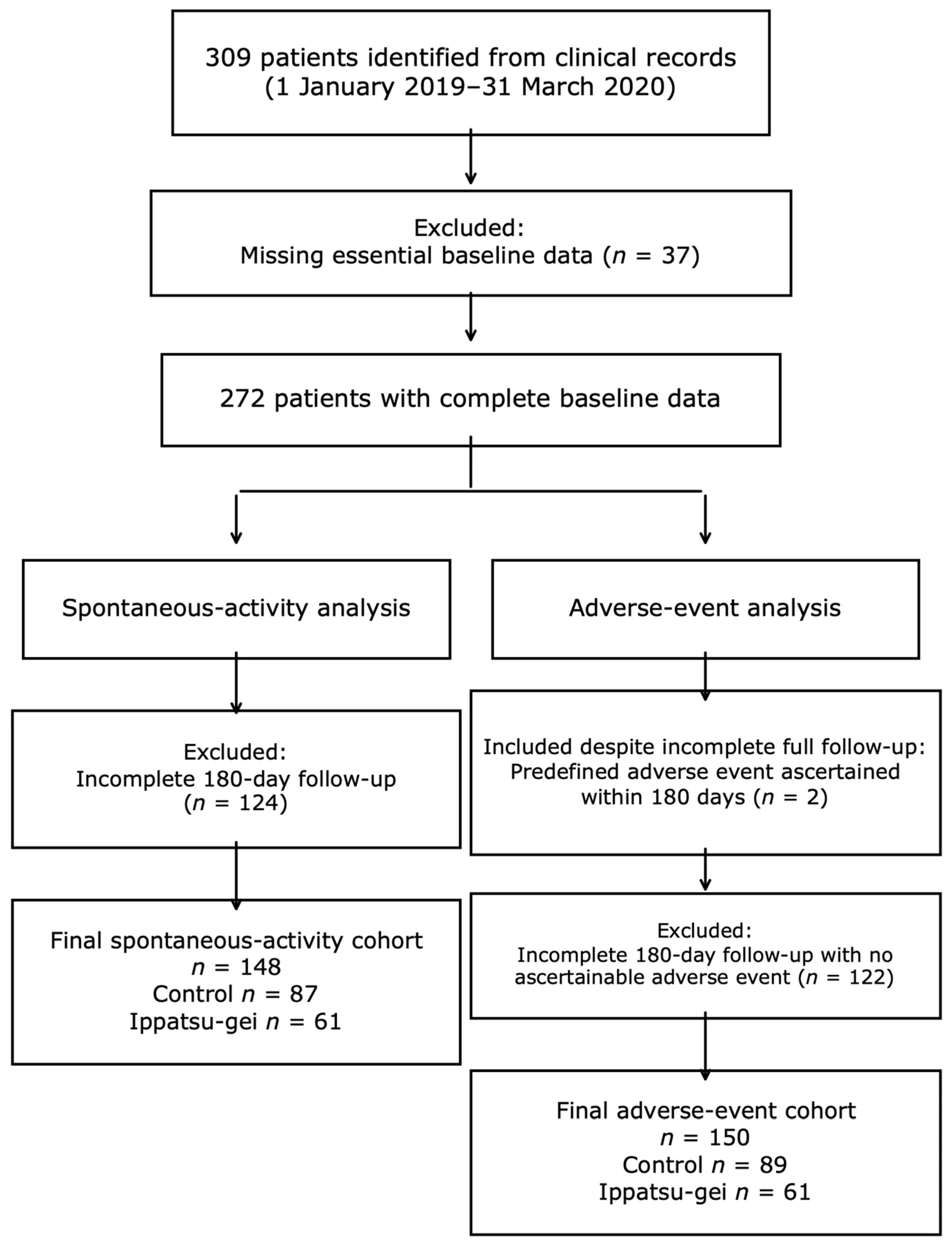
Flow diagram of patient inclusion and outcome-specific analytic cohorts. Clinical records were reviewed from 1 January 2019 to 31 March 2020. The spontaneous-activity analysis required complete 180-day follow-up. For the adverse-event analysis, two patients with a predefined adverse event ascertained within 180 days were retained despite incomplete 180-day follow-up, resulting in an adverse-event analytic cohort of 150 patients.

For the spontaneous-activity analysis, 124 patients without confirmed complete 180-day follow-up were excluded, resulting in an analytic cohort of 148 patients (87 in the control group and 61 in the Ippatsu-gei group). For the adverse-event analysis, two patients with predefined adverse events that were ascertained within 180 days were retained despite incomplete 180-day follow-up. Accordingly, 122 patients with incomplete follow-up and no ascertainable adverse event were excluded, resulting in an adverse-event analytic cohort of 150 patients (89 in the control group and 61 in the Ippatsu-gei group) (Figure 1).

Baseline demographic and clinical characteristics of the spontaneous-activity analytic cohort are summarized in Table 1. The age range of the overall cohort was 2–102 years. Several baseline characteristics showed notable imbalances between groups. Compared with the control group, the Ippatsu-gei group had a higher prevalence of limited oral intake (FOIS ≤3: 85.2% vs. 60.9%; SMD = 0.57), a lower prevalence of dementia (4.9% vs. 23.0%; SMD = 0.54), and a higher prevalence of physical injury (18.0% vs. 2.3%; SMD = 0.54). Prior rehabilitation exposure duration was substantially longer in the Ippatsu-gei group (median 28.1 [IQR 13.1–31.7] months) than in the control group (7.4 [1.5–15.6] months; SMD = 0.93). Other baseline differences are presented in Table 1. The distribution of spontaneous-activity subtypes is presented in Table S1.

### 3.2. Spontaneous Activity

During the 180-day observation period, spontaneous activity was documented in 18 of 148 patients (12.2%), including 5 of 87 patients (5.7%) in the control group and 13 of 61 patients (21.3%) in the Ippatsu-gei group.

In the univariate logistic regression analysis, Ippatsu-gei exposure was associated with higher odds of documented spontaneous activity (OR 4.44; 95% CI 1.49–13.23; *p* = 0.007). The association remained statistically significant after multivariable adjustment for age, sex, CFS, and prior rehabilitation exposure duration (aOR 5.42; 95% CI 1.62–18.18; *p* = 0.006) (Table 2). The confidence interval was wide, indicating substantial imprecision in the estimated magnitude of the association. None of the other covariates showed statistically significant associations with spontaneous activity.

In the sensitivity analysis using Firth’s penalized logistic regression, Ippatsu-gei exposure remained significantly associated with spontaneous activity (aOR 4.74; 95% CI 1.57–16.10; *p* = 0.005). The estimate was similar in direction to that obtained in the primary multivariable analysis, although the confidence interval remained wide (Table S2).

### 3.3. Adverse Events

During the 180-day observation window, adverse events were documented in 31 of 150 patients (20.7%), including 23 of 89 patients (25.8%) in the control group and 8 of 61 patients (13.1%) in the Ippatsu-gei group.

In the univariate logistic regression analysis, no statistically significant association was observed between Ippatsu-gei exposure and adverse events (OR 0.43; 95% CI 0.18–1.05; *p* = 0.063). In the multivariable logistic regression model adjusted for age, sex, and prior rehabilitation exposure duration, Ippatsu-gei exposure was not significantly associated with adverse events (aOR 0.53; 95% CI 0.20–1.39; *p* = 0.195) (Table 3). None of the other covariates showed statistically significant associations with adverse events.

In the sensitivity analysis using Firth’s penalized logistic regression, no statistically significant association between Ippatsu-gei exposure and adverse events was observed (aOR 0.55; 95% CI 0.20–1.37; *p* = 0.201) (Table S2). The wide confidence intervals indicate substantial uncertainty around the magnitude of the association.

## 4. Discussion

### 4.1. Principal Findings

In this retrospective cohort study, provider-based exposure to Ippatsu-gei during home-visit dysphagia rehabilitation was associated with higher odds of retrospectively documented spontaneous activity over a 180-day observation period. However, the estimated magnitude of this association was imprecise, as reflected by the wide confidence interval, and only 18 spontaneous-activity events were observed. Firth’s penalized logistic regression yielded an estimate in the same direction as the primary multivariable analysis, although the confidence interval remained wide.

No statistically significant association was observed between Ippatsu-gei exposure and adverse events. This finding should not be interpreted as evidence of safety or the absence of increased risk because the confidence intervals were wide and clinically relevant differences cannot be excluded.

These findings should therefore be interpreted as observational associations rather than evidence that Ippatsu-gei itself caused subsequent behavioral change. In particular, exposure was defined by contact with providers who routinely incorporated Ippatsu-gei into patient communication, and the temporal sequence between individual Ippatsu-gei interactions and spontaneous activity could not always be established.

### 4.2. Potential Mechanisms Underlying the Association Between Ippatsu-Gei Exposure and Spontaneous Activity

The observed association may be interpreted in the context of psychosocial and participation-oriented rehabilitation frameworks. Previous studies have linked activity and participation with rehabilitation and well-being in older adults [6,7], while spontaneity has also been examined in relation to psychological well-being [8]. Humor, laughter, and culturally meaningful activities have been investigated in relation to well-being and social functioning among older adults [10,11,12], while laughter and social communication have also been examined in relation to oral frailty [13]. Interpersonal trust has been examined as an important component of patient–provider relationships [14]. In addition, music- and singing-based approaches have been investigated in swallowing rehabilitation, although these interventions differ substantially from the informal Ippatsu-gei exposure examined in the present study [9].

In chronic home-visit dysphagia rehabilitation, many patients have substantial functional limitations, frailty, and restricted oral intake. In the present cohort, 70.9% had FOIS scores ≤3, and among participants who underwent VE, 36.7% had PAS scores of 6–8. In such settings, rehabilitation goals may extend beyond physiological aspects of swallowing to include maintenance of activity and participation, consistent with the ICF framework [15].

Ippatsu-gei in the present study consisted of brief, culturally familiar interpersonal interactions, including humor, songs, gestures, origami, and simple magic demonstrations. Such interactions could plausibly relate to rapport, emotional engagement, or willingness to participate in everyday activities. However, motivation, self-efficacy, emotional response, rapport, and behavioral activation were not directly measured in the present study. These pathways should therefore be regarded as possible explanations for the observed association rather than mechanisms demonstrated by the present findings.

Furthermore, because exposure classification was provider-based, the observed association may partly reflect differences in communication style, therapeutic relationships, clinical practice, or other characteristics of the providers who routinely used Ippatsu-gei, rather than the effect of Ippatsu-gei itself.

### 4.3. Adverse Events

No statistically significant association between Ippatsu-gei exposure and adverse events was observed during the 180-day observation period. The point estimate was below 1.0, but the confidence interval was wide, and the findings are compatible with a range of possible associations. Accordingly, clinically relevant differences in adverse-event occurrence cannot be excluded.

A strong direct association between Ippatsu-gei exposure and adverse events was not necessarily expected because Ippatsu-gei was a brief interpersonal interaction rather than an additional medical or rehabilitative procedure. Therefore, it may have had little direct influence on major medical events such as unplanned hospitalization, unplanned outpatient visits, or death. In addition, such adverse events are likely influenced by underlying disease burden, frailty, and other health-related factors that were not fully captured in the present analyses.

However, the limited number of events and the observational design preclude conclusions regarding safety. These findings indicate only that no statistically significant association with adverse events was detected in this cohort.

### 4.4. Clinical and Practical Implications

The present findings suggest that psychosocial and interpersonal aspects of routine home-visit dysphagia rehabilitation warrant further investigation alongside conventional physiological and functional approaches. The observed association with spontaneous activity raises the possibility that brief, culturally familiar interpersonal interactions may be relevant to patient engagement in daily life.

However, because Ippatsu-gei was not standardized and exposure was provider-based, the present findings do not justify recommending Ippatsu-gei as a specific therapeutic intervention. Rather, they highlight a broader research question concerning whether relational and context-sensitive elements of rehabilitation may be associated with activity and participation.

Although Ippatsu-gei is culturally specific to Japan, similar principles of brief, context-sensitive interpersonal engagement may take different forms across cultural settings. Future studies are needed to determine whether such approaches are reproducibly associated with patient-centered outcomes when evaluated prospectively and systematically.

### 4.5. Limitations

This study has several limitations. First, it was a single-center retrospective study, which limits generalizability and precludes causal inference.

Second, exposure classification was provider-based rather than based on systematic session-level documentation of individual Ippatsu-gei interactions. Consequently, the observed association cannot be disentangled from provider-specific characteristics, including communication style, therapeutic relationships, clinical judgment, or other aspects of care. The content, timing, frequency, and duration of Ippatsu-gei were also not standardized, precluding assessment of a dose–response relationship.

Third, spontaneous activity was retrospectively identified from routine clinical records using an operational definition rather than a validated instrument. Outcome review and classification were performed by a single investigator who was aware of exposure status. Therefore, underreporting, subjective classification, observer bias, and differential outcome ascertainment between exposure groups cannot be excluded.

Fourth, exposure and spontaneous activity were assessed during the same 180-day observation period, and the session-level timing of Ippatsu-gei was not systematically recorded. Therefore, the temporal sequence between exposure and spontaneous activity could not always be established. This limitation precludes determining whether Ippatsu-gei preceded the documented behavioral change.

Fifth, substantial baseline imbalance was observed between groups, particularly in prior rehabilitation exposure duration, FOIS, dementia, and physical injury. Although prior rehabilitation exposure duration and selected clinically relevant covariates were included in the multivariable models, the limited number of outcome events prevented adjustment for all observed baseline differences. Residual confounding from both measured and unmeasured factors therefore remains possible.

Sixth, only 18 spontaneous-activity events were observed, yielding wide confidence intervals and limited precision of the estimated association. Firth’s penalized logistic regression was used as a sensitivity analysis to reduce potential small-sample bias, but it does not eliminate the uncertainty associated with the small number of events.

Seventh, incomplete follow-up substantially reduced the analytic cohort. Complete 180-day follow-up was required for the spontaneous-activity analysis, whereas two patients with predefined adverse events ascertained within 180 days were retained in the adverse-event analysis despite incomplete follow-up. Exclusion of patients with incomplete follow-up may have introduced selection bias and may also have reduced representativeness and statistical power.

Eighth, severe frailty was highly prevalent, with CFS ≥6 in 98.0% of the spontaneous-activity cohort. This limited variability prevented stable simultaneous estimation of frailty and exposure effects in the adverse-event model and reduced the precision of risk estimates.

Finally, the study period was limited to the pre-COVID-19 period. Clinical records were reviewed through 31 March 2020, because the COVID-19 pandemic subsequently altered routine home-visit dental practice, including appointment cancellations and changes in care delivery. The cohort included both patients newly initiating home-visit dysphagia rehabilitation and those already receiving ongoing care at the index visit. Although this design allowed evaluation under relatively stable routine-care conditions, it differs from a conventional incident-cohort design and further limits causal interpretation.

### 4.6. Future Directions

Future studies should employ prospective, multicenter designs with systematic session-level documentation of Ippatsu-gei exposure, including timing, content, frequency, and duration. Recording the timing of both exposure and outcomes would allow temporal relationships to be evaluated more clearly and would help distinguish provider effects from the effects of the interaction itself.

Behavioral outcomes should also be assessed using standardized and validated instruments whenever possible. Objective measures, such as wearable activity monitoring, step counts, time spent out of bed, or other sensor-based measures of daily activity, could complement clinical-record-based assessments. Prospective assessment by investigators blinded to exposure status could further reduce observer and ascertainment bias.

Future studies should also consider provider-level factors, larger sample sizes, and analytic approaches that account for clustering by provider. Multicenter studies including culturally adapted forms of brief interpersonal engagement may help determine whether the observed association is reproducible across different clinical and cultural settings. Qualitative interviews with patients, caregivers, and clinicians may also clarify how such interactions are perceived and whether rapport, motivation, or emotional engagement mediate any association with activity and participation.

## 5. Conclusions

In this retrospective observational study, provider-based exposure, defined as care from dentists who routinely incorporated Ippatsu-gei into patient communication, was associated with retrospectively documented spontaneous activity during the 180-day observation period. However, the estimate was imprecise, and temporal uncertainty, provider-related effects, non-blinded outcome assessment, and residual confounding preclude causal interpretation. No statistically significant association with adverse events was observed. Prospective studies with standardized exposure documentation and objective outcome assessment are needed to better characterize these associations.

## Figures and Tables

**Table 1:** Baseline demographic and clinical characteristics of the primary spontaneous-activity analytic cohort (*N* = 148).

Variable	Control (*n* = 87)	Ippatsu-Gei (*n* = 61)	Total (*n* = 148)	SMD
Age, years, mean ± SD (range)	70.6 ± 23.5 (3–99)	64.0 ± 20.9 (2–102)	67.9 ± 22.6 (2–102)	0.297
Sex (male), *n* (%)	39 (44.8%)	35 (57.4%)	74 (50.0%)	0.253
Residence (home), *n* (%)	73 (83.9%)	53 (86.9%)	126 (85.1%)	0.084
CCI, mean ± SD	5.3 ± 2.7	5.5 ± 2.2	5.4 ± 2.5	0.079
Frailty (CFS ≥6), *n* (%)	85 (97.7%)	60 (98.4%)	145 (98.0%)	0.047
FOIS ≤3, *n* (%)	53 (60.9%)	52 (85.2%)	105 (70.9%)	0.570
FOIS 4–6, *n* (%)	34 (39.1%)	8 (13.1%)	42 (28.4%)	0.619
FOIS 7, *n* (%)	0 (0.0%)	1 (1.6%)	1 (0.7%)	0.183
PAS 1 (among participants with VE), *n* (%)	36/67 (53.7%)	15/31 (48.4%)	51/98 (52.0%)	0.107
PAS 2–5 (among participants with VE), *n* (%)	9/67 (13.4%)	2/31 (6.5%)	11/98 (11.2%)	0.235
PAS 6–8 (among participants with VE), *n* (%)	22/67 (32.8%)	14/31 (45.2%)	36/98 (36.7%)	0.255
JCS = 0, *n* (%)	71 (81.6%)	43 (70.5%)	114 (77.0%)	0.263
JCS I, *n* (%)	10 (11.5%)	13 (21.3%)	23 (15.5%)	0.267
JCS II, *n* (%)	5 (5.7%)	4 (6.6%)	9 (6.1%)	0.034
JCS III, *n* (%)	1 (1.1%)	1 (1.6%)	2 (1.4%)	0.042
Nervous system disease, *n* (%)	32 (36.8%)	18 (29.5%)	50 (33.8%)	0.155
Cerebrovascular disease, *n* (%)	24 (27.6%)	27 (44.3%)	51 (34.5%)	0.353
Dementia, *n* (%)	20 (23.0%)	3 (4.9%)	23 (15.5%)	0.540
Physical injury, *n* (%)	2 (2.3%)	11 (18.0%)	13 (8.8%)	0.539
Prior rehabilitation exposure duration, months, median [IQR]	7.4 [1.5–15.6]	28.1 [13.1–31.7]	12.9 [3.0–29.9]	0.931

Continuous variables are presented as mean ± standard deviation (SD) or median [interquartile range, IQR], as appropriate. Categorical variables are expressed as numbers (percentages). Residence was categorized as home or long-term care facility. Frailty was defined as a Clinical Frailty Scale score ≥6. Limited oral intake was defined as a Functional Oral Intake Scale score ≤3. FOIS scores of 1–3 indicate severe oral intake limitation, scores of 4–6 indicate partial oral intake, and a score of 7 indicates total oral intake with no restrictions. Levels of consciousness were classified using the Japan Coma Scale (JCS) as follows: JCS 0, alert; JCS I, awake without stimulus; JCS II, arousable with stimulation; and JCS III, unresponsive to stimulation. Prior rehabilitation exposure duration was defined as the number of months between the first-ever home-visit dysphagia rehabilitation and the index visit in 2019. All baseline characteristics were assessed at the index visit. PAS (Penetration–Aspiration Scale) data were available only for participants who underwent videoendoscopic evaluation of swallowing (VE) during routine clinical assessment. Absolute standardized mean differences (SMDs) are presented to describe baseline imbalance between groups; for prior rehabilitation exposure duration, the SMD was calculated from the underlying continuous values using means and standard deviations, although the variable is summarized in the table as median [IQR]. CCI, Charlson Comorbidity Index; CFS, Clinical Frailty Scale; FOIS, Functional Oral Intake Scale; IQR, interquartile range; JCS, Japan Coma Scale; PAS, Penetration–Aspiration Scale; SD, standard deviation; SMD, standardized mean difference; VE, videoendoscopic evaluation of swallowing.

**Table 2:** Associations between spontaneous activity and Ippatsu-gei exposure (*N* = 148).

Variable	Univariate OR (95% CI)	*p*-Value	Adjusted OR (95% CI)	*p*-Value
Ippatsu-gei (yes vs. no)	4.442 (1.492–13.226)	*p* = 0.007 *	5.420 (1.616–18.179)	*p* = 0.006 *
Age (years)	0.989 (0.970–1.010)	*p* = 0.304	0.990 (0.968–1.012)	*p* = 0.363
Sex (male)	1.000 (0.373–2.680)	*p* = 1.000	0.722 (0.249–2.095)	*p* = 0.549
CFS	0.719 (0.294–1.757)	*p* = 0.469	0.626 (0.233–1.680)	*p* = 0.352
prior rehabilitation exposure duration	1.009 (0.972–1.047)	*p* = 0.638	0.982 (0.942–1.025)	*p* = 0.416

Values are odds ratios (95% CI). * *p* < 0.05. CFS, Clinical Frailty Scale; CI, confidence interval; OR, odds ratio.

**Table 3:** Associations between adverse events and Ippatsu-gei exposure (*N* = 150).

Variable	Univariate OR (95% CI)	*p*-Value	Adjusted OR (95% CI)	*p*-Value
Ippatsu-gei (yes vs. no)	0.433 (0.179–1.046)	0.063	0.526 (0.199–1.390)	0.195
Age (years)	1.011 (0.992–1.031)	0.253	1.008 (0.988–1.028)	0.435
Sex (male)	0.891 (0.404–1.966)	0.776	1.015 (0.448–2.300)	0.971
prior rehabilitation exposure duration	0.975 (0.945–1.007)	0.128	0.987 (0.953–1.023)	0.476

Values are odds ratios (95% CI). CFS, Clinical Frailty Scale; CI, confidence interval; OR, odds ratio. The adjusted model included Ippatsu-gei exposure, age, sex, and prior rehabilitation exposure duration. When frailty (CFS) was included in exploratory models, convergence could not be achieved owing to sparse data and extreme coefficient estimates; therefore, results from this specification are not presented.

## Data Availability

Data cannot be publicly shared because of institutional and ethical restrictions. De-identified data are available upon reasonable request from the Ethics Committee of the Institute of Science Tokyo (email: rinri.adm@tmd.ac.jp).

## Supplementary Materials

The following supporting information can be downloaded at https://www.mdpi.com/article/10.3390/jcm15197745/s1, Table S1: Types of first documented spontaneous activity among patients with spontaneous activity (180-day cohort, *n* = 18); Table S2: Sensitivity analyses using Firth’s penalized logistic regression; Checklist S1: STROBE checklist for observational studies.

## Abbreviations

The following abbreviations are used in this manuscript:

VE	videoendoscopic evaluation of swallowing
ICF	International Classification of Functioning, Disability and Health
CFS	Clinical Frailty Scale
FOIS	Functional Oral Intake Scale
JCS	Japan Coma Scale
PAS	Penetration–Aspiration Scale
OR	odds ratio
CI	confidence interval
aOR	adjusted odds ratio

## Appendix A

### Appendix A.1. Outcome Definitions and Analytical Considerations

#### Appendix A.1.1. Definition of Spontaneous Activity

Spontaneous activity was operationally defined as newly emerging, patient-initiated behaviors documented in routine clinical records during the 180-day observation period following the index visit. These behaviors were not prescribed as part of the rehabilitation program and included going out of the home, initiating exercise, eating out, traveling, and increased time spent out of bed. Each patient was counted once regardless of the number or type of spontaneous activities documented. The outcome was treated as a binary variable indicating the presence or absence of any documented spontaneous activity. This record-based outcome was not a formally validated measure of motivation or spontaneity.

Spontaneous-activity events were identified and classified retrospectively according to this operational definition. Outcome review and classification were performed by a single investigator (C.K.), who was aware of Ippatsu-gei exposure status at the time of assessment. Therefore, outcome assessment was not blinded to exposure status.

#### Appendix A.1.2. Definition of Adverse Events

Adverse events were defined as the occurrence of unplanned hospitalization, unplanned outpatient visits, or death within 180 days of the index visit. Adverse events were treated as a binary outcome indicating the presence or absence of at least one predefined event during the observation period.

#### Appendix A.1.3. Follow-Up and Outcome Assessment

The index visit was defined as the first home-visit dysphagia rehabilitation session during the eligibility period from 1 January to 30 June 2019.

For the spontaneous-activity analysis, complete 180-day follow-up from the index visit was required because incomplete follow-up would limit the opportunity to ascertain whether spontaneous activity occurred during the full observation period. Patients without confirmed complete 180-day follow-up were therefore excluded from this analysis.

For the adverse-event analysis, patients were included if they either had confirmed complete 180-day follow-up or experienced a predefined adverse event that was ascertained within 180 days of the index visit before follow-up ended. Accordingly, patients with a confirmed adverse event within 180 days were classified as having experienced the outcome even when complete 180-day follow-up was unavailable. Patients with incomplete follow-up and no ascertainable adverse event were excluded because the absence of an adverse event could not be established throughout the entire 180-day observation period.

Because Ippatsu-gei exposure and spontaneous activity were both assessed during the same 180-day observation period and the timing of individual Ippatsu-gei interactions was not systematically documented at the session level, the temporal sequence between exposure and spontaneous activity could not always be established.

#### Appendix A.1.4. Exposure Classification

Exposure to Ippatsu-gei was provider-based rather than defined at the level of individual rehabilitation sessions. Patients were classified as exposed if they received at least one home-visit dysphagia rehabilitation session during the observation period from dentists who routinely incorporated brief entertainment-based psychosocial interactions into patient communication. Patients who did not receive care from such providers during the observation period were classified as unexposed.

Ippatsu-gei was not a standardized medical or dysphagia rehabilitation procedure. Its content, timing, frequency, and duration varied according to the clinical context and the treating dentist’s judgment and were not systematically recorded. Therefore, exposure classification indicates contact with providers who routinely used Ippatsu-gei and does not indicate that a standardized Ippatsu-gei interaction occurred during every rehabilitation session.

The observed associations may therefore partly reflect provider-related characteristics, such as communication style, therapeutic relationships, clinical judgment, or other aspects of routine practice, rather than the effect of Ippatsu-gei itself.

### Appendix A.2. Supplementary Statistical Analysis

Logistic regression analyses were performed using IBM SPSS Statistics for Windows, version 29.0 (IBM Corp., Armonk, NY, USA). Univariate analyses were first conducted to examine associations between Ippatsu-gei exposure and each outcome.

Multivariable models included covariates selected a priori based on clinical relevance, existing literature, and model stability considerations. Because the number of outcome events was limited, model complexity was restricted.

For spontaneous activity, the multivariable model included Ippatsu-gei exposure, age, sex, Clinical Frailty Scale (CFS) score, and prior rehabilitation exposure duration. Only 18 spontaneous-activity events were observed, and therefore the resulting estimates should be interpreted with consideration of the limited event count and wide confidence intervals.

For adverse events, the multivariable model included Ippatsu-gei exposure, age, sex, and prior rehabilitation exposure duration. Severe frailty was highly prevalent, and inclusion of CFS in the adverse-event model resulted in unstable estimation with non-estimable coefficients. CFS was therefore not included in the final adverse-event model.

Several baseline characteristics, including prior rehabilitation exposure duration, Functional Oral Intake Scale score, dementia, and physical injury, differed between exposure groups. Because of the limited number of outcome events, not all observed baseline differences could be included simultaneously in the multivariable models. Residual confounding therefore cannot be excluded.

Odds ratios (ORs) and adjusted odds ratios (aORs) were reported with 95% confidence intervals (CIs). A two-tailed *p*-value < 0.05 was considered statistically significant.

As a sensitivity analysis, Firth’s penalized logistic regression was performed using the same covariate specifications as the primary multivariable models to reduce potential small-sample bias associated with the limited number of outcome events. These analyses were conducted using R version 4.6.1 (R Foundation for Statistical Computing, Vienna, Austria) with the logistf package (version 1.26.1). Adjusted odds ratios with 95% profile penalized likelihood confidence intervals and corresponding *p*-values are presented in Table S2.

No correction for multiple comparisons was applied because the analyses were hypothesis-driven and exploratory.

#### Notes on Interpretation

The analyses in this study were observational and exploratory. Associations between provider-based Ippatsu-gei exposure and study outcomes should not be interpreted as causal effects. In particular, potential provider-related confounding, uncertainty regarding the temporal sequence between exposure and spontaneous activity, non-blinded retrospective outcome assessment, and residual confounding should be considered when interpreting the findings. The absence of a statistically significant association with adverse events should not be interpreted as evidence of safety.
