# Supplementary material for: Association of “Ippatsu-Gei,” a Brief Psychosocial Interaction, with Spontaneous Activity and Adverse Events in Home-Visit Dysphagia Rehabilitation: A Retrospective Cohort Study
Source: J Clin Med. 2026 Oct 8;15(19):7745. doi: 10.3390/jcm15197745 (PMC13665812; doi:10.3390/jcm15197745)
Supplement: Supplementary file 1 [file jcm-15-07745-s001.zip › jcm-4571400-supplementary.pdf]

Supplementary Material

Table S1. Types of first documented spontaneous activity among patients with spontaneous activity (180-day cohort, n = 18)

Activity	Control (n = 5)	Ippatsu-gei (n = 13)	Total (n = 18)
Going out	2	7	9
Exercise initiation	2	3	5
Eating out	1	1	2
Travel	0	1	1
Increased time out of bed	0	1	1

Activities were identified retrospectively from routine clinical records during the 180-day observation period following the index visit. Each patient was counted once and assigned to the category corresponding to the first documented spontaneous activity. All patients with documented spontaneous activity (Control, n = 5; Ippatsu-gei, n = 13; Total, n = 18) are included in this table.

Table S2. Sensitivity analyses using Firth's penalized logistic regression

**Outcome/Variable**	**aOR**	**95% CI**	**p-value**
**Spontaneous activity**			
Ippatsu-gei exposure	**4.74**	**1.57–16.10**	**0.005**
Age	0.99	0.97–1.01	0.332
Sex (male)	0.73	0.26–2.04	0.551
CFS	0.60	0.26–1.62	0.265
Prior rehabilitation exposure duration	0.98	0.94–1.02	0.424
**Adverse events**			
Ippatsu-gei exposure	**0.55**	**0.20–1.37**	**0.201**
Age	1.01	0.99–1.03	0.486

Sex (male)	1.01	0.45–2.26	0.983
Prior rehabilitation exposure duration	0.99	0.95–1.02	0.484

Values are adjusted odds ratios (aORs) with 95% profile penalized likelihood confidence intervals. Firth's penalized logistic regression was performed using the same covariate specifications as the primary multivariable models. The spontaneous-activity model (N = 148) was adjusted for age, sex, CFS, and prior rehabilitation exposure duration; the adverse-event model (N = 150) was adjusted for age, sex, and prior rehabilitation exposure duration. CFS, Clinical Frailty Scale; CI, confidence interval; aOR, adjusted odds ratio.

Checklist S1. STROBE checklist for observational studies

The completed STROBE checklist is provided below. Locations refer to sections, tables, figures, and appendices in the main manuscript.

Item No.	Section/Topic	STROBE Recommendation	Reported in Manuscript (Location)
1a	Title and abstract	Indicate the study design with a commonly used term in the title or the abstract.	Title; Abstract
1b	Title and abstract	Provide in the abstract an informative and balanced summary of what was done and what was found.	Abstract
2	Background/rationale	Explain the scientific background and rationale for the investigation being reported.	Section 1, Introduction
3	Objectives	State specific objectives, including any prespecified hypotheses.	Section 1, final paragraph
4	Study design	Present key elements of study design early in the paper.	Section 2.1, Study design and setting

Item No.	Section/Topic	STROBE Recommendation	Reported in Manuscript (Location)
5	Setting	Describe the setting, locations, and relevant dates, including periods of recruitment, exposure, follow-up, and data collection.	Sections 2.1–2.2; Appendix A.1
6a	Participants	Give the eligibility criteria, and the sources and methods of selection of participants. Describe methods of follow-up.	Section 2.2; Figure 1; Appendix A.1
6b	Participants	For matched studies, give matching criteria and numbers of exposed and unexposed.	Not applicable; no matching was performed
7	Variables	Clearly define all outcomes, exposures, predictors, potential confounders, and effect modifiers. Give diagnostic criteria, if applicable.	Sections 2.3–2.5; Appendix A.1
8	Data sources/measurement	For each variable of interest, give sources of data and details of methods of assessment.	Sections 2.3–2.5; Appendix A.1
9	Bias	Describe any efforts to address potential sources of bias.	Sections 2.2, 2.5, and 2.6; Section 4.5
10	Study size	Explain how the study size was arrived at.	Section 2.2; all eligible patients during the defined eligibility period were screened; Figure 1
11	Quantitative	Explain how quantitative variables were handled in the	Sections 2.4 and 2.6

Item No.	Section/Topic	STROBE Recommendation	Reported in Manuscript (Location)
	variables	analyses. If applicable, describe which groupings were chosen and why.	
12a	Statistical methods	Describe all statistical methods, including those used to control for confounding.	Section 2.6; Appendix A.2
12b	Statistical methods	Describe any methods used to examine subgroups and interactions.	Not applicable; no subgroup or interaction analyses were performed
12c	Statistical methods	Explain how missing data were addressed.	Sections 2.2 and 2.6; missing data were not imputed; Figure 1
12d	Statistical methods	Explain how loss to follow-up was addressed.	Section 2.2; Appendix A.1; Figure 1; complete 180-day follow-up was required for the spontaneous-activity analysis, whereas patients with a predefined adverse event ascertained within 180 days were retained in the adverse-event analysis despite incomplete 180-day follow-up
12e	*Statistical methods*	*Describe any sensitivity analyses.*	Section 2.6; Appendix A.2; Table S2
13a	Participants	Report numbers of individuals at each stage of the study.	Section 3.1; Figure 1

Item No.	Section/Topic	STROBE Recommendation	Reported in Manuscript (Location)
13b	Participants	Give reasons for non-participation at each stage.	Section 3.1; Figure 1
13c	Participants	Consider use of a flow diagram.	Figure 1
14a	Descriptive data	Give characteristics of study participants and information on exposures and potential confounders.	Section 3.1; Table 1
14b	Descriptive data	Indicate the number of participants with missing data for each variable of interest.	Section 3.1; Figure 1; Table 1
14c	Descriptive data	Summarize follow-up time.	Sections 2.2 and 3.1; outcome-specific 180-day observation procedures described in Appendix A.1
15	Outcome data	Report numbers of outcome events or summary measures over time.	Sections 3.2–3.3; Tables 2–3; Table S1
16a	Main results	Give unadjusted and adjusted estimates and their precision. Make clear which confounders were adjusted for and why they were included.	Sections 2.6 and 3.2–3.3; Tables 2–3; Appendix A.2
16b	Main results	Report category boundaries when continuous variables were categorized.	Section 2.4; clinical thresholds are defined where categorical variables are reported
16c	Main results	If relevant, consider translating estimates of relative risk into absolute risk for a meaningful time period.	Sections 3.2–3.3; absolute event counts and percentages are reported for the 180-day observation period

Item No.	Section/Topic	STROBE Recommendation	Reported in Manuscript (Location)
17	Other analyses	Report other analyses performed, including subgroup, interaction, and sensitivity analyses.	Sections 3.2–3.3; Appendix A.2; Table S2
18	Key results	Summarize key results with reference to study objectives.	Section 4.1, Principal findings
19	Limitations	Discuss limitations of the study, taking into account sources of potential bias or imprecision.	Section 4.5, Limitations
20	Interpretation	Give a cautious overall interpretation of results considering objectives, limitations, multiplicity, similar studies, and other relevant evidence.	Sections 4.1–4.5; Section 5, Conclusions
21	Generalisability	Discuss the generalisability (external validity) of the study results.	Sections 4.4–4.5
22	Funding	Give the source of funding and the role of the funders for the present study and, if applicable, the original study.	Funding statement; no external funding
